# Ultrasound-guided treatment of giant isolated gastric varices with gastric-renal shunt using coil, cyanoacrylate, and titanium clips

**DOI:** 10.1055/a-2719-3285

**Published:** 2025-11-10

**Authors:** Yuchuan Bai, Zhihong Wang, Yaxian Kuai, Xuecan Mei, Derun Kong

**Affiliations:** 1 36639 Department of Gastroenterology, The First Affiliated Hospital of Anhui Medical University, Hefei, China

In cases where isolated gastric varices (IGV) are complicated by gastrorenal shunt (GRS), endoscopic injection of cyanoacrylate (CYA) carries a risk of ectopic embolism, especially when the GRS diameter is 1 cm or greater 1 . Balloon-occluded retrograde transvenous obliteration (BRTO) is one of the most commonly used methods for reducing ectopic embolism 2 3 . However, after reducing the lumen of IGV, the placement of coils with an appropriate diameter under endoscopic ultrasonography is also a viable option.

A 71-year-old male was initially diagnosed with alcoholic cirrhosis (Child-Pugh class A). Preoperative abdominal CTA revealed a GRS measuring 10.6 mm ( Fig. 1 ). Direct endoscopy revealed a large IGV ( Fig. 2 **a** ), with a maximum diameter of approximately 3.5 cm measured. Two titanium clips (ROCC-F-26-195, Micro-Tech (Nanjing) Co., Ltd) were applied to clamp the vessel ( Fig. 2 **b** ). The maximal vascular diameter measured by EUS (EG-580UT; Fujifilm) was 2.1 cm, whereas the diameter at the titanium clip site measured only 1 cm ( Fig. 2 **c** ). Two coils (M0013120660, Boston Scientific) were placed under EUS guidance ( Fig. 2 **d** , Video 1 ), followed by CYA injection. Under direct endoscopy, vascular lesions with palpable softness were treated with CYA injection (DEI-CYA) ( Fig. 2 **e** ). EUS was used again to observe, and the vascular lumen was completely occluded ( Fig. 2 **f** ). Follow-up CTA showed the disappearance of varices and extramural vessels, with the GRS still present ( Fig. 3 ), and no clinical symptoms of ectopic embolism were observed. The patient did not experience bleeding or other complications 1 month postoperatively.

**Fig. 1:**
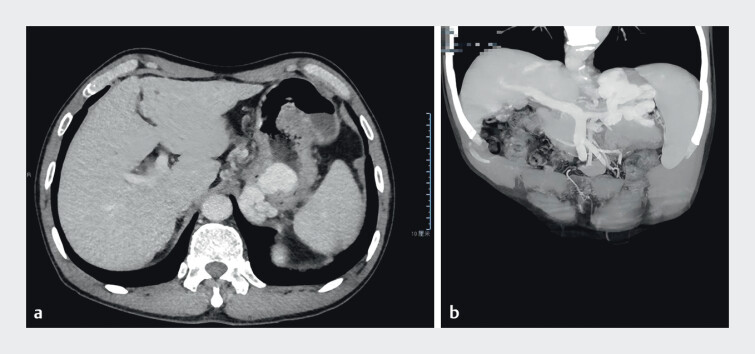
Preoperative abdominal and pelvic CTA revealed a massive variceal mass in the gastric fundus, which was connected to extramural vessels, indicating the presence of GRS.

**Fig. 2:**
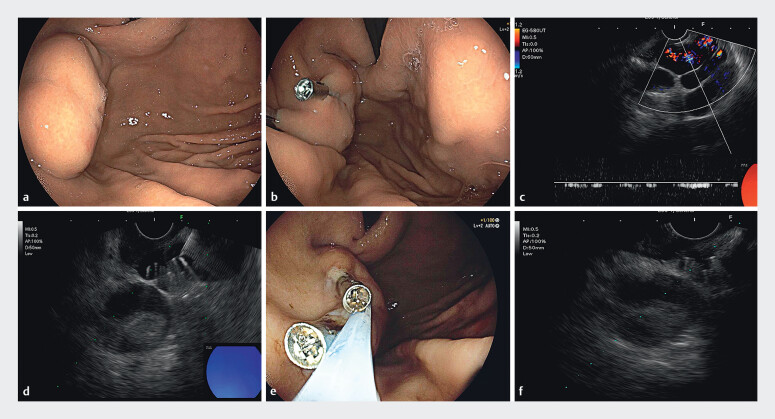
**a** Direct endoscopy revealed a large tumor-like IGV; **b** Under direct endoscopy, titanium clips were observed blocking the blood vessel; **c** After titanium clips treatment, EUS measurement showed that the diameter of the blood vessel was approximately 21.56 mm; **d** EUS revealed a high-echoic shadow in the shape of “∞”; **e** DEI-CYA; **f** The varicose veins disappeared after treatment.

**Fig. 3:**
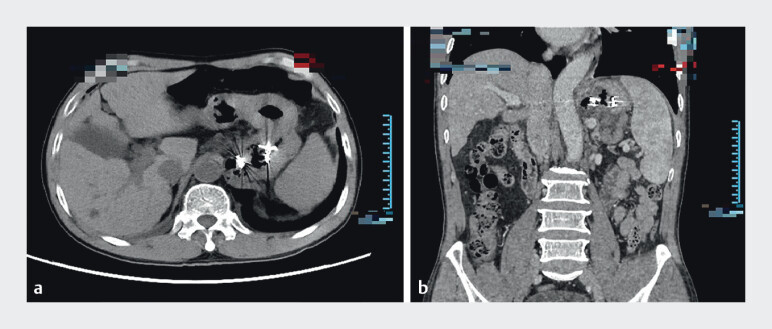
Follow-up abdominal and pelvic CTA after treatment showed the disappearance of varices and extramural vessels, with the GRS still present.

A giant IGV is visible under white-light endoscopy. First, some blood vessels are clipped with titanium clips, and then spring coil-tissue adhesive treatment is administered under EUS guidance.Video 1

In this case, we reduced the diameter of IGV using titanium clips and combined coil placement with CYA injection to avoid ectopic embolism. For vessels that were difficult to inject under EUS, DEI-CYA treatment was combined to achieve complete occlusion of the large variceal mass. Postoperative CTA also confirmed the success of this treatment in this case.

Endoscopy_UCTN_Code_TTT_1AS_2AB

## Notes

Endoscopy E-Videos https://eref.thieme.de/e-videos
*E-Videos* is an open access online section of the journal *Endoscopy* , reporting on interesting cases and new techniques in gastroenterological endoscopy. All papers include a high-quality video and are published with a Creative Commons CC-BY license. Endoscopy E-Videos qualify for HINARI discounts and waivers and eligibility is automatically checked during the submission process. We grant 100% waivers to articles whose corresponding authors are based in Group A countries and 50% waivers to those who are based in Group B countries as classified by Research4Life (see: https://www.research4life.org/access/eligibility/ ). This section has its own submission website at https://mc.manuscriptcentral.com/e-videos .
